# High-Intensity Interval Training in Patients with Substance Use Disorder

**DOI:** 10.1155/2014/616935

**Published:** 2014-03-02

**Authors:** Grete Flemmen, Runar Unhjem, Eivind Wang

**Affiliations:** ^1^Department of Circulation and Medical Imaging, Faculty of Medicine, The Norwegian University of Science and Technology, 7006 Trondheim, Norway; ^2^Department of Research and Development, Clinic of Substance Use and Addiction Medicine, St.Olav's University Hospital, 7030 Trondheim, Norway

## Abstract

Patients with substance use disorder (SUD) suffer a higher risk of cardiovascular disease and other lifestyle diseases compared to the general population. High intensity training has been shown to effectively reduce this risk, and therefore we aimed to examine the feasibility and effect of such training in SUD patients in clinical treatment in the present study. 17 males and 7 females (32 ± 8 yr) in treatment were randomized to either a training group (TG), treadmill interval training in 4 × 4 minutes at 90–95% of maximal heart rate, 3 days a week for 8 weeks, or a conventional rehabilitation control group (CG). Baseline values for both groups combined at inclusion were 44 ± 8 (males) and 34 ± 9 (females) mL *·* min^−1^ *·* kg^−1^, respectively. 9/12 and 7/12 patients completed the TG and CG, respectively. Only the TG significantly improved (15 ± 7%) their maximal oxygen consumption (VO_2max_), from 42.3 ± 7.2 mL *·* min^−1^ *·* kg^−1^ at pretest to 48.7 ± 9.2 mL *·* min^−1^ *·* kg^−1^ at posttest. No between-group differences were observed in work economy, and level of insomnia (ISI) or anxiety and depression (HAD), but a significant within-group improvement in depression was apparent for the TG. High intensity training was feasible for SUD patients in treatment. This training form should be implemented as a part of the rehabilitation since it, in contrast to the conventional treatment, represents a risk reduction for cardiovascular disease and premature death.

## 1. Introduction

Patients with substance use disorder (SUD), classified within ICD-10: F10-19 (mental and behavioral disorders due to psychoactive substance use) at the World Health Organization's mental and behavioral disorders classification, have a high prevalence of health and psychosocial problems in addition to their substance use disorder [1]. Although this patient group's disorder indeed has multifactorial causes, the evidence of how their physical capacity may be related to their calamitous lifestyle is sparse. Contributing to a decreased life expectancy of 15–20 years, the lowest among patients with different mental illnesses [2, 3] is an increased prevalence of cardiovascular disease [4–6]. The high risk of developing cardiovascular disease is associated with the population's drug use, poor nutrition, and obesity but is also likely a direct result of the patient group's inactivity [3].

Endurance training, especially with emphasis on high intensity, is shown to increase aerobic power and reduce the risk of cardiovascular disease [7–10]. Improvements of 10–30% in maximal oxygen consumption (VO_2max⁡_) have typically been observed in these studies, after training interventions of 2-3 months. These improvements may also be associated with large reductions in the risk of mortality, as an improvement of 1MET (~3.5 mL · min⁡^−1^ · kg^−1^) is shown to reduce the mortality rate by 12% [11]. Adding to the physical benefits of exercise are also possible effects on mental health. Although little is known about the effects of high-intensity interval training in SUD patients, exercise has been documented to have an overall beneficial effect on mental health and quality of life in patients with mental illnesses [12].

Despite the well-documented effect on cardiovascular disease risk reduction [7, 8, 10], decreased mortality rate [11, 13, 14], and improved mental health [15–19], effective physical training appears not to be a part of the conventional treatment for SUD patients. There has certainly been physical activities in the clinics for more than 30 years [20], but in general this activity seems random and unstructured and the frequency and intensity of the activities are often unknown. Indeed the activities may vary between clinics, but to our knowledge, it has not been documented that whole body exercise with a high intensity (≥85% of HR_max⁡_) constitutes a part of the clinical program. As physiological parameters rarely are documented, knowledge of physical status or improvements remains uncertain. Therefore, the aim of the present study was to examine if high-intensity interval training was feasible for SUD patients in treatment. Further, we aimed to document their aerobic power and compare the training group, if they were able to adhere, with patients receiving conventional treatment in the same clinic. Our hypotheses were that SUD patients (1) are able to complete a high-intensity interval training program, (2) have a decreased aerobic power at baseline compared to the average population, and (3) improve their VO_2max⁡_ and work economy more than the control group receiving conventional rehabilitation.

## 2. Methods

### 2.1. Subjects

**Figure 1:**
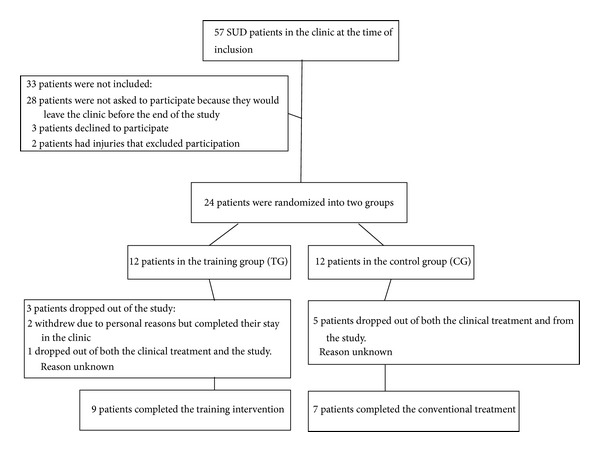
Recruitment, randomization, and withdrawal of SUD patients throughout the study.

24 patients with a diagnosis of substance use disorder, ICD-10: F10-F19, were included in this study. All subjects participated in residential long term treatment in a substance abuse treatment clinic at the time of the study, due to abuse of illegal drugs. The long term treatment program at the clinic lasts for ~3 months. Subjects were excluded if they had been abstinent or/and systematically participated in endurance training for the last six months. Subjects were also excluded if they had cardiovascular disease or chronic obstructive pulmonary disease or were not able to perform treadmill testing and training. After signing the written informed consent, patients were randomized to either a high intensity training group (TG) or a conventional rehabilitation control group (CG) (Figure 1). Patient characteristics and medical use are given in Table 1. The regional ethical committee did approve the study, and it was carried out in accordance with the Declaration of Helsinki.

### 2.2. Testing

#### 2.2.1. Maximal Oxygen Consumption and Work Economy

Measurements of VO_2max⁡_, work economy, and ventilatory parameters were obtained using the Cortex Metamax II portable metabolic test system (Cortex Biophysik GmbH, Leipzig, Germany), walking/running on a treadmill (Woodway Weil am Rhein, Germany). After a 10-minute warm-up period, the subjects walked at 4.5 km · h^−1^ at 5% inclination for a period of 5 minutes. The average oxygen consumption for the last minute of this period was recorded as the work economy. Immediately following the work economy test, the subjects continued to the VO_2max⁡_ test. The incline was kept at 5% while velocity was increased by 1 km · h^−1^ every minute until exhaustion. VO_2max⁡_, respiratory exchange ratio (RER) and ventilation were calculated averaging the three highest continuous 10 second values. One or more of the following criteria for reaching VO_2max⁡_ were considered [21]: (1) if the oxygen consumption reached a plateau despite further increases in workload, (2) a RER above 1.05, and (3) lactate concentration in blood ([La^−^]_b_) > 7 mmol. Maximal heart rate (HR_max⁡_) was calculated as 4 beats · min^−1^ added to the highest heart rate during the last minute [22]. For heart rate assessment Polar F6 heart rate monitors were used (Polar Electro, Finland). [La^−^]_b_ were measured using the Biosen C_line (EKF Diagnostics GmbH, Barleben, Germany) analyzer. Blood from the patient's fingertip was sampled for analysis of blood lactate within 1 min after the VO_2max⁡_ test. As an expression of maximal aerobic power, inclination and velocity at VO_2max⁡_ were registered.

#### 2.2.2. Identification of Drug Use

For identification of the extent of drug use the first page of EuropASI was applied (Addiction Severity Index, European adaption of The American 5th edition [23]). This index quantifies which substances have been used, when the patients started their use, and for how long the dependency has lasted. Further, the medical use for the patients participating in the study is given in Table 1.

#### 2.2.3. Insomnia, Anxiety, and Depression Questionnaires

In addition to the physical testing two questionnaires were implemented; Insomnia Severity Index (ISI) to detect possible cases of insomnia and Hospital Anxiety and Depression Scale (HAD) which is used to estimate the levels of anxiety and depression. These self-report questionnaires were answered before and after the training intervention as measures of psychological changes during the period of the study. The ISI has been evaluated to be a clinically useful tool for screening and quantifying perceived insomnia severity [24]. It is composed of 7 items targeting different categories of sleep disturbance severity. The items are rated at a five-point Likert scale (0–4) summed up to a total score ranging from 0 to 28, where a higher score indicates more severe insomnia. The score categories are 0–7 (no clinically significant insomnia), 8–14 (subthreshold insomnia), 15–21 (clinical insomnia, moderate severity), and 22–28 (clinical insomnia, severe). The HAD self-assessment scale is consisting of a fourteen item scale, seven items related to anxiety and seven related to depression. On the subscales for anxiety and depression a score of 0–7 for either subscale is estimated within the normal range, while a score of 11 or higher implies a probable presence of a mood disorder. A score of 8–10 is considered signs of a mood disorder [25].

### 2.3. Training Intervention

Both the TG and the CG participated in the clinic treatment activities throughout the 8-week intervention period. These activities included: Ballgames (indoor-soccer and volleyball), yoga, stretching, outdoor walking, low resistance strength training, ceramics, TV games, and card games. Additionally,the TG received supervised training 3 times a week for a period of 8 weeks. The training was performed as inclined walking or running on a treadmill, using the same heart rate monitor as during the VO_2max⁡_ testing, to ensure correct intensity of every training session. The training sessions were organized as interval training, with 4 × 4 minutes of high aerobic intensity (90–95% of HR_max⁡_), interrupted by 3-minute recovery periods (~70% of HR_max⁡_) [21]. All training sessions were supervised. As the subjects improved, velocity and incline were increased to meet the targeted heart rate. The subjects needed to have an adherence of at least 20 out of 24 training sessions in order to be included in the data analyses. Within the same time period as the TG performed their high-intensity interval training on a treadmill, the patients in the CG chose to participate in a self-elected activity among the offered sports or games in the clinical treatment program. Although representing a wide range of different activities, they all shared a measured or estimated intensity level of <70% of HR_max⁡_.

### 2.4. Statistics

Statistical analyses were performed using the software SPSS, version 20 (Chicago, USA), and figures were made using the software GraphPad Prism 5 (San Diego, USA). Relative improvements are given as mean percentage change. To determine if the data was normal distributed a Q-Q plot was used. Repeated measures ANOVAs (2 (group) × 2 training status) were used to determine differences between groups following training. If appropriate, a Tukey post hoc analysis was used. Unpaired and paired *t*-tests were used to detect differences between groups at baseline and within group following training, respectively. Statistical significance was accepted at an *α*-level of *P* < 0.05. Data are reported as mean ± SD, unless otherwise noted. Additionally, using similar statistics, an intention to treat analysis with the use of last observation carried forward for missing data was carried out for all the 24 subjects that were randomized to the two groups. To achieve a statistical power of 80%, 8 patients in each group needed to complete the study period in order to observe a 0.375 L · min⁡^−1^ improvement difference in mean VO_2max⁡_ between TG and CG, assuming a SD of 0.25 L · min⁡^−1^. These values were based on previous studies from our group using the same training intervention in other populations. The drop-out rate in previous studies has been ~2/10 subjects. However, considering that this patient population may be more challenging than average, a higher drop-out rate is expected. Thus 12 subjects were randomized to each group to ensure observation of the assumed efficacy difference between the two groups.

## 3. Results

11 of the 12 SUD patients in the TG and 7 out of 12 patients in the CG, respectively, completed their overall intended stay at the substance use disorder clinic. With regard to the high intensity training, 3 subjects withdrew from the TG. Two withdrew due to personal reasons but remained in the clinical treatment, while one dropped out from both the clinical treatment and the TG. In the CG 5 subjects dropped out of the clinical treatment and thus withdrew from the study without giving any reasons (Figure 1). The SUD patients that completed the training period carried out 22 ± 1 of the scheduled supervised training sessions. The targeted intensity (90–95% of HR_max⁡_) was reached in all completed sessions. None of the subjects reported any problems or discomfort completing the training sessions, other than the normal strain following high intensity exercise.

**Figure 2:**
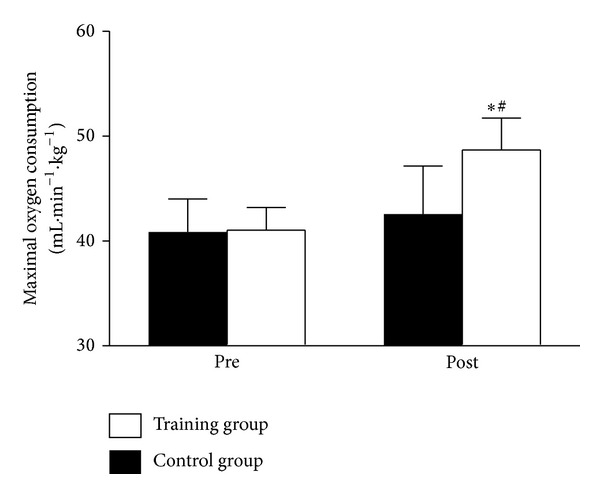
Maximal oxygen consumption before and after the training intervention. Data are presented as mean ± SE. *P < 0.01, difference within group from pre- to posttest, ^#^ *P* < 0.05, differences in changes from pre- to posttest between groups.

At baseline, before the withdrawal of subjects from the study, values for both groups combined were 44 ± 8 (males) and 34 ± 9 (females) mL · min⁡^−1^ · kg^−1^, respectively. VO_2max⁡_ significantly (*P* < 0.01) improved by 15 ± 7% for the 9 subjects that completed the TG (Table 2). This improvement was also significantly (*P* < 0.01) different from the CG (Figure 2). In accordance with the improvement in aerobic power, the TG also increased velocity and inclination at VO_2max⁡_ from 9.2 ± 2.3 km · h^−1^ and 5.6 ± 1.1% at pretest to 9.3 ± 2.2 km · h^−1^ and 8.3 ± 2.4% at posttest. The CG showed no within-group improvement in neither VO_2max⁡_ nor maximal workload. The TG increased ventilation at VO_2max⁡_ by 14 ± 10%, while there were no differences within or between groups in RER or [La^−^]_b_ at VO_2max⁡_ from pre- to posttest. An intention to treat analysis, including all 24 participants that were randomized to either the TG or the CG, did not show different results for the primary outcomes compared to analysis including only subjects that completed the study.

Work economy, measured at 5% inclination and 4.5 km · h^−1^ on the treadmill, showed no significant differences between or within the two groups following the training period (Table 3). However, the heart rate at the work economy workload significantly (*P* < 0.05) decreased by 9 ± 12% in the TG, but this within-group change was only apparent as a trend (*P* = 0.158) when compared to the CG.

For psychological variables, the TG displayed a significant (*P* < 0.05) decrease in depression level following the training period, whereas the CG had a significant decrease (*P* < 0.05) in anxiety level from pre- to posttest (Table 4). However, neither of these within-group differences, measured by the Hospital Anxiety and Depression questionnaire, was apparent as between-group differences.

## 4. Discussion

Since little is known about the aerobic power of SUD patients in treatment and their lifestyle indicates that they may suffer a high risk of cardiovascular and lifestyle diseases, this study sought to investigate the aerobic power of this group of patients and their response to exercise training of high intensity. The main findings of the study were as follows (1) the initial aerobic power at baseline is lower than what is typically seen in the average population, (2) the SUD patients improved their aerobic power and work performance following the training intervention, thus decreasing the risk factors for lifestyle diseases, and (3) the training intervention is applicable as a part of the clinical treatment.

### 4.1. Reduced Aerobic Power in Patients with Substance Use Disorder

At inclusion, the SUD patients in the present study had a baseline VO_2max⁡_ of 44 ± 8 (males) and 34 ± 9 (females) mL · min⁡^−1^ · kg^−1^. This is well below age-matched reference data from the average population [26]. The 10% and 16% lower baselines for the ~30 year old males and females, respectively, are comparable to the average values observed among 50–59-year-old healthy subjects [26]. The low aerobic power, as documented in the current study, is in line with a previous study displaying VO_2max⁡_ values of 39 (males) and 31 mL · min⁡^−1^ · kg^−1^ (females) in SUD patients [27]. Our study and the Mamen and Martinsen [27] study are to our knowledge the only studies to directly assess aerobic power in SUD patients. However, the assumption of a health-related critical low aerobic power is also supported by several studies applying estimations of VO_2max⁡_ [17, 28–31]. Since low aerobic power is a well-established risk factor for cardiovascular disease and all-cause mortality [11, 13, 14, 32], it is likely that the low VO_2max⁡_ observed among SUD patients may, at least in part, be responsible for the elevated prevalence of cardiovascular disease and premature death observed in this patient group. It is therefore surprising that aerobic power commonly is not listed as one of the major causes for illnesses, medical conditions, and early death in SUD patients [3, 4, 6].

### 4.2. Exercise-Induced Effect on Aerobic Power, Work Load, and Risk Reduction

As expected, the SUD patients that completed the supervised eight week treadmill training period in the current study improved VO_2max⁡_. In accordance with the 15 ± 7% improvement in VO_2max⁡_, the TG increased maximal velocity and inclination at VO_2max⁡_ from 9.2 ± 2.3 km · h^−1^ and 5.6 ± 1.1% at pretest to 9.3 ± 2.2 km · h^−1^ and 8.3 ± 2.4% at posttest. For patient groups with low aerobic capacities, daily activities are often perceived as strenuous. An improvement in work capacity is therefore typically associated with an increased wellbeing in everyday life, since it reduces the relative intensity on the daily tasks [10, 33, 34]. The improvement in VO_2max⁡_ observed in our study is similar to what have previously been reported following a whole body high intensity (>85% of HR_max⁡_) training intervention in a wide range of patient groups [7–10, 35, 36], as well as and in healthy subjects [21, 37] and old subjects [37]. The magnitude of VO_2max⁡_ improvement may be influenced by training status, age, or pathology [7–10]. Subjects with a low baseline are, both mathematically and physiologically, susceptible to larger percentage improvements (~15–35%) than subjects with a higher baseline (~6%–13%) [21, 37].

Considering the elevated risk of mortality [3] and cardiovascular incidents in SUD patients [4], VO_2max⁡_ improvements as demonstrated in the present study are beneficial. A 3.5 mL · min⁡^−1^ · kg^−1^ improvement in VO_2max⁡_ has been shown to be associated with a 12% improved chance of survival [11] and a 15% reduced risk for developing cardiovascular disease [32]. The SUD patients in the present study improved their VO_2max⁡_ by 6.6 mL · min⁡^−1^ · kg^−1^, indicating that not only will they have a strongly decreased mortality rate, but also a considerable reduced risk of developing cardiovascular disease. After the relatively short-duration training period the SUD patients restored their VO_2max⁡_ values to a level similar to the age-matched healthy population [26]. In contrast, it is thought provoking that conventional clinical treatment did not improve VO_2max⁡_. Physical activity is certainly applied in today's treatment [20], but clearly this physical activity is not sufficient to induce improvements in VO_2max⁡_. Since the conventional activities are all reported to be carried out with a low intensity, this may explain the lack of improvement, as intensity is suggested to be the key factor for VO_2max⁡_ improvements [21, 38]. Recognizing the high risk for cardiovascular disease and mortality in these patients, it is critical that today's treatment may have no effect on one of the most important factors for these conditions.

The exercise-induced improvement in VO_2max⁡_ that was observed in the TG is likely due to an improvement in maximal cardiac output, and more specific is the stroke volume of the heart since no changes were observed in HR_max⁡_. Previously the stroke volume has been shown to be the decisive factor that explains the adaptations to high intensity training, both in moderately trained healthy subjects [21] and in untrained coronary artery disease patients [39]. The ~42 mL · min⁡^−1^ · kg^−1^ (males and females combined) baseline VO_2max⁡_ in the current study falls between an aerobic power of ~55 (young, healthy) and ~27 (coronary artery disease) mL · min⁡^−1^ · kg^−1^. Although the SUD patients indeed represent a different group of subjects, it is likely, since the stroke volume adaptations appear to be similar across different populations, that also their improvements in VO_2max⁡_ originate from training-induced changes in maximal stroke volume.

### 4.3. Training Effect on Insomnia, Anxiety, and Depression

A positive relationship between exercise and mood disorders is well-documented [20, 40–44] specifically apparent as insomnia [40, 45], anxiety [44], and depression disorder [41, 43, 46] reductions. Therefore, it is surprising that the large difference between the TG and the CG in aerobic power and work load following the study period did not induce detectable differences between groups in any of these variables. At baseline, the level of mental distress in both the TG and the CG group was ranged as moderately severe according to the Insomnia Severity Index and the Hospital Anxiety and Depression Scale. Both groups scored within subthreshold for insomnia and within signs of mood disorder. Following the study period there was a reduction of the depression variable within the TG, as well as a reduction of the anxiety variable within the CG, but these reductions were not different between the two groups. It is possible that psychological benefits, as measured by the questionnaires in our study, are more related to physical activity per se and not necessarily to aerobic exercise training. Although reduced depression symptoms may be associated with exercise in general and not necessarily restricted to the aerobic form of exercise [15], it should undoubtedly be expected that a risk reduction of cardiovascular disease and mortality would cause an improvement of mood disorders and quality of life [42]. Thus the commonly applied questionnaires that were used in our study should be able to detect such an improvement. Our results indicate that a supplement, expansion, or replacement to/of the questionnaires are sought for, although it is recognized that the relatively small sample size in the current study may be, in part, responsible for the nondetectable differences in mood disorders.

### 4.4. High-Intensity Interval Training: Clinical Implications

Considering their high rate of nonattendance and discontinuation [47, 48], reflected in the high relapse rates from clinical treatment [49], an important question in the current study was whether the SUD patients were able to carry out the scheduled period of training. To our knowledge there have not been any previous reports of SUD patients participating in such an intensive training intervention. The SUD patients were in our study capable of managing the intensive training, reflected in the high attendance (22 ± 1 of the total 24 scheduled training sessions) for the 9 subjects that completed the training period. Interestingly, the completion rate was higher for the TG, both in the current study and in the clinical treatment, compared to the CG. Only 1 out of 12 patients in the TG dropped out from clinical treatment (5 subjects in the CG) and 3 from the training study (5 subjects in the CG). Certainly these withdrawals could be due to chance, but the completion rate is nevertheless a testament to the feasibility of high-intensity interval training as part of the clinical treatment. An intention to treat analysis showed no differences in the main findings of the current study and thus strengthens the result of an overall beneficial effectiveness of high-intensity interval training in a clinical setting. Not only was it feasible to apply this intervention in the clinic, but additionally the subjects that completed the training reported no discomfort or pain during the training sessions other than what should be expected with high intensity training, and not one single commenced training session was aborted.

Our findings are in line with a previous study applying a similar training intervention in patients with schizophrenia [10]. In the Heggelund et al. study [10] the training was reported to be challenging but feasible. In our study, as well as in the Heggelund et al. study [10], all trainings were conducted in presence of a supervisor. This may be favorable when implementing a training intervention in mentally ill patients. Mamen et al. [50] emphasized that the relationship between the patient and the supervisor can prove essential for the patients motivation and commitment to the project. Our experience throughout this study supports this notion. The present study exemplifies that high intensity training can be applicable for SUD patients in treatment and that the intensive training is manageable also for this patient group. Considering the health benefits associated with this training, it should be implemented as a complementary treatment for SUD patients.

## 5. Conclusion

In the present study SUD patients are shown to have a low aerobic power, and thus they are at risk for developing cardiovascular disease. As it is important that SUD patients receive both a physical and psychological treatments in the clinic and our results indicate that the conventional treatment is not sufficient to reduce the risk of cardiovascular disease, high-intensity interval training should be implemented as part of the clinical treatment to effectively improve the patient groups' aerobic power.

## Figures and Tables

**Table 1:** Patient characteristics and medical use.

	TG (*n* = 9)	CG (*n* = 7)	Combined (*n* = 16)
Men/women (*n*)	8/1	5/2	13/3
Age (yr)	33 ± 11	31 ± 8	32 ± 9
Height (cm)	177 ± 10	175 ± 10	176 ± 10
Weight (kg)	84.1 ± 14.9	87.9 ± 20.8	85.8 ± 17.2
Current smoker	8	6	14
Drug use debut (age)	15 ± 6	17 ± 4	16 ± 5
Duration of abuse (yr)	17 ± 8	12 ± 4	15 ± 7
Primary drug			
Heroin	1	1	2
BZD, Sed, Hypn	1	1	2
Amphetamine	4	4	8
Cannabis	3	1	4
Secondary drug			
Alcohol	2	0	2
Heroin	1	1	2
Opiates, painkillers	2	1	3
Amphetamine	1	0	1
Cannabis	3	5	8
Symptoms for medicine prescription:			
ADHD	0	1	1
Allergies	1	2	3
Anxiety	2	1	3
Arthritis	0	1	1
Asthma/COPD	0	1	1
Depression	1	2	3
Epilepsy	0	1	1
Hypertension	3	1	4
Schizophrenia/bipolar	1	2	3
Substitutional treatment	1	1	2
Other	0	3	3

Data are presented as mean ± SD; TG: training group; CG: control group, Type of medication is reported on indication of symptoms according to common directory. The prescribed medicines in substitutional treatment are methadone and suboxone. Others: skin disorder, pain, and inflammation.

**Table 2:** Changes in physiological parameters from pre- to posttest.

	TG (*N* = 9)		CG (*N* = 7)	
	Pre	Post	Pre	Post
VO_2max⁡_				
(L·min^−1^)	3.60 ± 0.91	4.15 ± 1.03^∗∗#^	3.43 ± 0.66	3.54 ± 0.65
(mL·kg^−1^·min^−1^)	42.3 ± 7.2	48.7 ± 9.2^∗∗#^	41.8 ± 12.3	42.6 ± 12.1
*V*_*E*_ (L·min^−1^)	109.2 ± 28.6	125.9 ± 36.8^∗∗#^	103.5 ± 24.4	103.2 ± 23.9
RER	1.09 ± 0.03	1.10 ± 0.02	1.17 ± 0.11	1.14 ± 0.06
HR_max⁡_ (beats·min^−1^)	180 ± 11	181 ± 14	189 ± 7	188 ± 7
[La^−^]_b_	9.12 ± 2.41	10.65 ± 1.69	8.04 ± 3.54	9.21 ± 2.01

Data are presented as mean ± SD. TG: training group; CG: control group; VO_2max⁡_: maximal oxygen uptake; *V*_*E*_: ventilation; RER: respiratory exchange ratio; HR_max⁡_: maximal heart rate; [La^−^]_b_: lactate concentration in blood, **P < 0.01, difference within group from pre- to posttest, ^#^ *P* < 0.05, differences in changes from pre- to posttest between groups.

**Table 3:** Work economy measured at 4.5 km·h^−1^ and 5% inclination at pre- and posttest.

	TG (*N* = 9)		CG (*N* = 7)	
	Pre	Post	Pre	Post
VO_2_				
(L·min^−1^)	1.54 ± 0.24	1.61 ± 0.28	1.70 ± 0.36	1.77 ± 0.43
(ML·kg^−1^·min^−1^)	18.5 ± 1.1	18.9 ± 1.3	19.6 ± 2.3	20.2 ± 1.4
*V*_*E*_ (L·min^−1^)	35.1 ± 4.4	33.3 ± 5.3	38.3 ± 9.3	41.1 ± 9.0
RER	0.90 ± 0.04	0.89 ± 0.08	0.93 ± 0.05	0.94 ± 0.07
HR_max⁡_ (beats·min^−1^)	116 ± 17	105 ± 18	116 ± 18	119 ± 9

Data are presented as mean ± SD. TG: training group; CG: control group; VO_2_: oxygen uptake; *V*_*E*_: ventilation; RER: respiratory exchange ratio; HR_max⁡_: maximal heart rate.

**Table 4:** Psychological changes from pre- to posttest (scores from the insomnia severity index and hospital anxiety and depression scale questionnaires).

	TG (*n* = 9)		CG (*n* = 7)	
	Pre	Post	Pre	Post
Anxiety	9.4 ± 3.5	8.6 ± 2.5	9.1 ± 5.3	6.3 ± 3.4*
Depression	8.5 ± 4.8	5.3 ± 3.9*	6.0 ± 3.5	4.6 ± 3.5
Insomnia	10.6 ± 5.4	8.9 ± 5.1	10.9 ± 10.1	9.1 ± 5.0

Data are presented as means ± SD. TG: training group; CG: control group. *P < 0.05, difference within group from pre- to posttest.
